# The Potential Alleviating Property Against NAFLD by *Ganoderma lucidum* With Bacteria‐Enzyme Synergistic Fermentation

**DOI:** 10.1002/fsn3.71981

**Published:** 2026-06-02

**Authors:** Manhui Sun, Kecheng Li, Zhenzhen Wang, Jing Dai, Jianwei Mao, Min Cai, Ruyi Sha

**Affiliations:** ^1^ School of Biological and Chemical Engineering, Zhejiang University of Science and Technology Hangzhou China; ^2^ Zhejiang Provincial Key Laboratory for Chemical & Biological Processing Technology of Farm Product Hangzhou China

**Keywords:** antioxidant activity, fermentation, ganoderic acid, ganoderma, NAFLD, network pharmacology

## Abstract

*Ganoderma lucidum* is an edible and medicinal fungus. Triterpenoids, particularly ganoderic acids, represent the principal bioactive constituents in *Ganoderma lucidum*. Bacteria‐enzyme synergistic fermentation broth of *Ganoderma lucidum* (FBG) was optimized as a potential alternative to traditional water‐extracted *Ganoderma lucidum* (WEG). The optimized parameters with *Lactobacillus rhamnosus* fermentation exhibited the highest content of ganoderic acid F (GAF). Administration of FBG led to a pronounced decline in lipid deposition and contents of intracellular total cholesterol (TC), triglycerides (TG), low‐density lipoprotein cholesterol (LDL‐C), reactive oxygen species (ROS), and malonic dialdehyde (MDA). The FBG treatment also markedly increased the activities of high‐density lipoprotein cholesterol (HDL‐C), superoxide dismutase (SOD), and catalase (CAT). The results of network pharmacology analysis and molecular docking suggested that GAF exhibits a robust interaction with the core targets sodium leak channel, nonselective (NALCN) and eukaryotic translation elongation factor 1 alpha 2 (EEF1A2). Enzyme‐linked immunosorbent assay (ELISA) results demonstrated that GAF could effectively reduce lipid accumulation while upregulating EEF1A2, phosphorylated AMP‐activated protein kinase (p‐AMPK), and peroxisome proliferator‐activated receptor alpha (PPAR‐α) protein expression. Collectively, these findings indicate that GAF alleviates free fatty acid (FFA)‐induced hepatic lipid accumulation through activation of the EEF1A2/p‐AMPK/PPAR‐α signaling pathway, suggesting a potential mechanism involving AMPK‐related pathways.

## Graphical abstract

A bacteria‐enzyme synergistic fermentation process using *Lactobacillus rhamnosus* was developed to produce fermented *Ganoderma lucidum* broth (FBG), which significantly enriched ganoderic acid F compared to water extraction. In FFA‐induced HepG2 cells, FBG alleviated NAFLD‐related damage by reducing lipid deposition, ROS, MDA, TC, TG, and LDL‐C, while increasing HDL‐C, SOD, and CAT activities. Network pharmacology and molecular docking identified NALCN and EEF1A2 as direct targets of ganoderic acid F. Mechanistically, ganoderic acid F upregulated EEF1A2, enhanced AMPK phosphorylation, and induced PPAR‐α expression, thereby activating the EEF1A2/AMPK/PPAR‐α pathway to suppress hepatic lipid accumulation. These findings support the potential alleviating property of fermented *Ganoderma lucidum* and ganoderic acid F against NAFLD, highlighting them as promising therapeutic strategies.

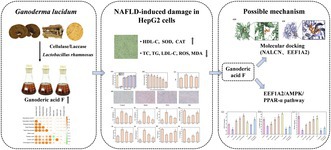

**Abbreviations**
AMPK: AMP‐activated protein kinase
AUC: area under the curve
BCA: bicinchoninic acid
CAT: catalase
DEGs: differentially expressed genes
DMEM: Dulbecco's modified Eagle medium
DMSO: dimethyl sulfoxide
EEF1A2: eukaryotic translation elongation factor 1 alpha 2
ELISA: enzyme‐linked immunosorbent assay
FBG: bacteria‐enzyme synergistic fermentation broth of *Ganoderma lucidum*
FBS: fetal bovine serum
FFA: free fatty acid
GAF: ganoderic acid F
GO: gene ontology
HDL‐C: high‐density lipoprotein cholesterol
HPLC: high‐performance liquid chromatography
KEGG: Kyoto encyclopedia of genes and genomes
LASSO: least absolute shrinkage and selection operator
LDL‐C: low‐density lipoprotein cholesterol
MDA: malonic dialdehyde
MTT: methylthiazolydiphenyl‐tetrazolium bromide
NAFLD: nonalcoholic fatty liver disease
NALCN: sodium leak channel, nonselective
p‐AMPK: phosphorylated AMP‐activated protein kinase
PBS: phosphate buffer saline
PPAR‐α: peroxisome proliferator‐activated receptor alpha
PPI: protein–protein interaction
RF: random forest
ROC: receiver operating characteristic curve
ROS: reactive oxygen species
RSM: response surface methodology
SOD: superoxide dismutase
TC: intracellular total cholesterol
TG: triglycerides
WEG: traditional water‐extracted *Ganoderma lucidum*

## 1 Introduction

*Ganoderma lucidum* (*Reishi*) is an edible and medicinal fungus that has been used in China as a medicinal fungus with health‐preserving and healthcare effects for over 2000 years (Cui et al. 2015). The fruiting body of *Ganoderma* consists of a pileus, stipe, hymenium, and context, which contain bioactive compounds such as polysaccharides (Daoust et al. 2025). Currently, only *Ganoderma* spore powder is utilized in industry, while the fruiting bodies are largely underutilized or discarded (Sheng et al. 2020). Additionally, the physical methods used for breaking *Ganoderma* spore walls often involve high temperatures, which lead to a significant reduction in the content of bioactive components (Zhao et al. 2014). Traditional processing methods of *Ganoderma* fruiting bodies include slicing, crushing for brewing, or extracting active compounds for use in capsules and other health products. According to a previous study, the major bioactive components of *Ganoderma* are primarily located within the cell walls (Chen et al. 2025). However, the high content of cellulose and lignin in the cell wall makes the degradation process more difficult, thus limiting the release of bioactive components and reducing the bioavailability of these compounds. Since cellulase and laccase can degrade cellulose and lignin, respectively, they disrupt the rigid structure of the cell wall. Therefore, microbial fermentation utilizing these enzymes presents a promising strategy to enhance the release and bioavailability of bioactive compounds from *Ganoderma* (Wang et al. 2025). It is a mild processing method that effectively preserves the integrity of bioactive compounds (Sun et al. 2023). In addition, microbial fermentation significantly enhances the bioavailability of active ingredients (Markkinen et al. 2022) through the metabolic utilization of polysaccharides and other nutrients (Hur et al. 2014). This process not only promotes the release of additional bioactive compounds (Boateng et al. 2024) but also contributes to transforming macromolecules with low biological activity into highly active small molecular entities (Wu et al. 2024) and may even lead to the generation of novel bioactive substances (Guo et al. 2025).

The chemical constituents of *Ganoderma* (Gong et al. 2019) include triterpenoids, meroterpenoids, steroids, alkaloids, nucleosides, bases, and polysaccharides (Lu et al. 2020). A variety of pharmacological activities have been observed in these compounds, such as anti‐inflammatory, immunomodulatory (Wu et al. 2025), anti‐tumor, hepatoprotective, hypoglycemic, and hypotensive effects. Especially, triterpenoids play a crucial role in regulating cellular metabolism and exhibit high antioxidant activity by effectively scavenging intracellular free radicals. They can also maintain the integrity and fluidity of cell membranes, and support normal physiological metabolic processes (Liu et al. 2025). Ganoderic acids, a class of lanostane‐type triterpenoids found in *Ganoderma* species, are among the most important pharmacologically active compounds in *Ganoderma lucidum* and exhibit antioxidant, anti‐neuropsychiatric, anti‐tumor, and immunomodulatory effects (Song et al. 2025). Nearly 40 types of ganoderic acids have been identified, including ganoderic acid A, F, B, and D, among others. The composition and proportion of these ganoderic acids in *Ganoderma* fruiting bodies vary depending on species, growth stage, and specific fruiting body parts (Tian et al. 2024). Other bioactive components of *Ganoderma*, such as polysaccharides, ganoderic acids, and total phenols, have been shown to down‐regulate plasma triglyceride and total cholesterol (TC) levels (Gao et al. 2024). They were also reported to regulate lipid metabolism by reducing intracellular triglyceride and cholesterol contents, thereby alleviating fat accumulation and preventing lipid metabolism‐related disorders (Zhang et al. 2025).

Nonalcoholic fatty liver disease (NAFLD) is a spectrum of metabolic disorders closely associated with obesity (Seo et al. 2023), ranging from simple hepatic steatosis to nonalcoholic steatohepatitis (NASH), and can progress to fibrosis, cirrhosis, and hepatocellular carcinoma (Lu et al. 2025). NAFLD is strongly linked to dysregulated lipid metabolism and elevated oxidative stress (Liang et al. 2022), and its global prevalence has been steadily increasing, posing a growing public health burden (Le et al. 2023). Current NAFLD treatments may cause side effects and metabolic challenges. For instance, ezetimibe improves liver fibrosis but elevates hemoglobin A1c (HbA1c) and hepatic long‐chain fatty acids (Powell et al. 2023). Additionally, NAFLD reduces drug‐metabolizing enzyme expression; cytochrome P450 2C19 (CYP2C19) is notably downregulated in NASH patients, slowing drug clearance and reducing the efficacy of CYP2C19‐activated drugs (Takeshita et al. 2014). Therefore, targeting lipid metabolism and reducing oxidative stress remain key strategies for NAFLD management (Kuang et al. 2023; Wang et al. 2015).

This study investigated the dynamic changes of key bioactive components (ganoderic acids, organic acids, total phenols, and flavonoids) in Ganoderma fermentation broth. The potential of this fermented broth in alleviating NAFLD was evaluated through in vitro assays and mechanistic studies. Candidate genes were screened using NAFLD‐related sequencing data from public databases, followed by functional enrichment analysis (gene ontology (GO) and Kyoto Encyclopedia of Genes and Genomes (KEGG)) (Hu et al. 2024). Hub genes were identified using least absolute shrinkage and selection operator (LASSO) regression and random forest algorithms, and their binding affinity with ganoderic acid F (GAF) was assessed via molecular docking. This study provides a scientific basis for the development of fermented Ganoderma products as potential interventions for NAFLD.

## 2 Materials and Methods

### 2.1 Production of WEG and FBG

Traditional water‐extracted *Ganoderma lucidum* (WEG): *Ganoderma lucidum* fruiting bodies were milled and filtered using a 20‐mesh sieve (Kuang et al. 2023). The sample was mixed with ultrapure water and heated in a 90°C water bath for 1 h, then cooled and filtered. The extract was collected and stored for later use (Hu et al. 2024).

Bacteria‐enzyme synergistic fermentation broth of *Ganoderma lucidum* (FBG): The sample of *Ganoderma lucidum* fruiting body powder was placed in an erlenmeyer flask. Cellulase (0.8%), laccase (0.3%), and ultrapure water were added at a specified solid–liquid ratio. The blended mixture was sealed and enzymatically hydrolyzed by maintaining it at 50°C for 12 h. Post‐hydrolysis, sterilization was performed through autoclaving at 121°C for 15 min. After cooling, fermentation cultures were introduced, and incubation proceeded in a shaker set to 37°C and 150 rpm for the required fermentation time (Hu et al. 2024). Upon completion of fermentation, filtration was used to isolate the supernatant, which was preserved for further examination. Liquid‐state fermentations with different fermentation durations were conducted in independent systems, with three replicates per group.

GAF content served as the critical response variable for systematically evaluating the impact of key fermentation parameters on the *Ganoderma lucidum* bioprocess. For the effect of fermentation strains, a constant solid‐to‐liquid ratio of 1:25 was employed throughout the experiment. With 5% inoculation of each *Lactobacillus plantarum* , *Lactobacillus bulgaricus* , and *Lactobacillus rhamnosus* , fermentation time was set to 48 h. To evaluate fermentation duration, the ratio of solids to liquids was maintained at 1:25, inoculated with 5% *Lactobacillus rhamnosus* , and fermentation times tested were 0, 24, 48, 72, and 96 h. For the effect of bacterial inoculation amount, the solid–liquid ratio was 1:25, fermentation time 48 h, and *Lactobacillus rhamnosus* inoculation amounts tested were 4%, 5%, 6%, 7%, and 8%. For evaluating the effect of varying solid–liquid ratios, fermentation was conducted for 48 h with a fixed 6% inoculation of *Lactobacillus rhamnosus*, testing ratios of 1:20, 1:30, 1:40, 1:50, and 1:60 (Yuan et al. 2023). All experiments were performed in three independent biological replicates, each containing three technical replicates.

Response surface methodology (RSM) experiment (Ding et al. 2025): Building on the findings from single‐factor experiments, fermentation time, inoculum size, and solid–liquid ratio were chosen as variables for further analysis. A three‐factor, three‐level RSM design was constructed using Design‐Expert 13 software (Bagher 2025), with the content of GAF serving as the response variable. The specific factors and their corresponding levels are provided in Table S1.

### 2.2 High‐Performance Liquid Chromatography (HPLC) Test

Several ganoderic acids were quantified using HPLC. The mobile phase comprised methanol and water, with 0.1% formic acid added to the aqueous component (Kuang et al. 2023). Gradient elution was carried out following the protocol detailed in Table S2. Detection occurred at 254 nm wavelength while the column temperature remained constant at 40°C.

The three organic acids: malic acid, tartaric acid, and lactic acid were separated and performed using a mobile phase composed of methanol (Solvent A) and 0.01 mol/L KH_2_PO_4_ buffer (Solvent B, pH 2.7) in a 2:98 (v/v) ratio. For the determination of chlorogenic acid, the mobile phase comprised methanol (Solvent A) and distilled water (Solvent B) mixed in a 30:70 (v/v) proportion (Li et al. 2022). Both organic acids and chlorogenic acid were analyzed using isocratic elution with the column maintained at 25°C. An injection volume of 10 μL alongside a flow rate of 1.0 mL/min was consistently applied across all analyses (Kuang et al. 2023). Reference solutions for the compounds were prepared at a concentration of 1 mg/mL using methanol as the solvent. The calibration data derived from HPLC analysis can be found in Table S3. To guarantee sample purity, all solutions were filtered through a 0.22 μm organic membrane before testing (Yuan et al. 2023).

### 2.3 Viable Count and Physicochemical and Nutritional Properties Test

Viable count was assessed using the trypan blue exclusion assay. Briefly, cells were mixed with an equal volume of 0.4% trypan blue solution, loaded onto a hemocytometer, and viable (unstained) cells were counted under a light microscope (Subramanian et al. 2024).

Total phenolic concentration was determined via the folin–ciocalteu procedure, with flavonoid content measured (Kuang et al. 2023) by a sodium nitrite–aluminum nitrate‐based colorimetric assay following sample dilution (Li et al. 2022).

### 2.4 Cell Viability Assay

Cultured HepG2 cells were cultured according to reported protocols (Li et al. 2022) with dulbecco's modified eagle medium (DMEM) medium which contain 10% fetal bovine serum (FBS) and 1% antibiotics (Li et al. 2022) at 37°C with 5% CO_2_. At 60%–70% confluency, cells were rinsed with phosphate buffer saline (PBS), then treated with 0.25% trypsin for 1 min, and centrifuge at 1000 rpm for 5 min. Cells from the third stable passage were used for subsequent experiment.

After adjusting the cell density to 1 × 10^5^ cells/mL, cells suspension were inoculated into 96‐well plates with 100 μL per well. After 24 h preincubation, the culture medium was then discarded. For the sample groups, cells were treated with 100 μL of complete medium containing either WEG or FBG (54 h) with different dilution ratios (undiluted, 20×, 50×, 100×, 200×) or with various concentrations of GAF (1, 5, 10, 20, and 50 μmol/L). For the positive control group, cells were treated with 0.1 μmol/L atorvastatin (Solarbio, Beijing, China) (Cerda et al. 2021).

For the AMP‐activated protein kinase (AMPK) inhibitor group, cells were treated with 10 μmol/L Compound C (Solarbio, Beijing, China) (Fu et al. 2024). Since all the components to be tested were dissolved in dimethyl sulfoxide (DMSO), the final DMSO concentration in the culture medium was maintained at 0.5% for all groups (El‐Faham et al. 2015). All components were filter‐sterilized using a 0.22 μm membrane before application (Shi et al. 2025). For the blank control group, cells were treated with 100 μL fresh medium. All groups were incubated for 24 h.

Then cell viability was assessed by using the methylthiazolydiphenyl‐tetrazolium bromide (MTT) assay. Following 3 h incubation at 37°C with 50 μL MTT (0.5 mg/mL), formazan crystals in cells were dissolved in 150 μL DMSO, and absorbance was measured at 570 nm with a microplate reader (You et al. 2021). For WEG and FBG groups, the maximum concentration that showed no significant difference compared with the blank control group was selected for subsequent experiments. For GAF, all groups with no significant difference from the blank control were selected for further experiments.

### 2.5 Establishment of the NAFLD Model

Free fatty acid (FFA) preparation method: 9.19 mg of sodium palmitate (MW 278.41 g/mol) and 20.1 mg of sodium oleate (MW 304.44 g/mol) were each dissolved separately in 10 mL of deionized water. The solutions underwent sonication and were subsequently heated in a 65°C water bath. Then, they were mixed and diluted to a final concentration of 5 mmol/L, followed by storage at −20°C until further use (Hu et al. 2024).

Log‐phase HepG2 cells were inoculate in 96‐well plates at cell density of 1 × 10^5^ cells/mL. After preincubation for 24 h, the medium were discharged and cells were treated with FFA solutions at final concentrations of 0.1, 0.25, 0.5, 0.8, 1.0, and 2.0 mmol/L for 24 h (Frion‐Herrera et al. 2025). Then, cell viability was assessed by MTT method as mentioned in Section 2.4. FFA concentrations with no significant difference versus blank control were chosen for further experiments. All experiments were independently repeated three times as biological replicates, each with three technical replicates.

### 2.6 Oil Red O Staining

The Oil red O working solution was prepared at a 3:2 dilution of the 0.5% isopropanol stock solution with distilled water, filtered, and incubated in darkness for 10 min. After inducing steatosis, cells in 24‐well plates were washed with PBS and then treated with 200 μL Oil red O working solution for 20 min. After staining, cells were rinsed twice with PBS. Intracellular lipids were then extracted with 600 μL of 70% ethanol for 20 min at room temperature. The extracted solution (200 μL) was transferred to a 96‐well plate, and lipid content was quantified at 490 nm.

### 2.7 Assay of Intracellular TC, TG, LDL‐C, HDL‐C

HepG2 cells were inoculated in 6‐well plates at 1 × 10^5^ cells/mL (2 mL per well, *n* = 3 replicates). At the end of the experiment, cells were washed with PBS and lysed with 500 μL buffer for 30 min. Lysates were centrifuged (12,000 rpm, 4°C, 5 min) and the supernatants were assayed for intracellular TC, triglycerides (TG), low‐density lipoprotein cholesterol (LDL‐C), and high‐density lipoprotein cholesterol (HDL‐C) (Matsumoto et al. 2024) by commercial kits and normalized by the bicinchoninic acid (BCA) method (Bagher 2025).

### 2.8 Assay of Intracellular ROS and Antioxidant Enzymes

HepG2 cells were inoculated in 24‐well plates for reactive oxygen species (ROS) determination. At the end of the experiment, the culture medium was discarded, and 1 mL of 2′,7′‐Dichlorodihydrofluorescein diacetate (DCFH‐DA) fluorescent probe was added to each well, followed by incubation for 30 min (Zhang et al. 2022). Then, cells were rinsed with serum‐free medium and PBS‐replenished for ROS quantification per kit specifications. Posttreatment, cells were PBS‐washed and lysed with 200 μL radio‐immunoprecipitation assay (RIPA) lysis buffer. The SOD and CAT activities of collected lysates were analyzed by commercial kits and normalized by the BCA method.

### 2.9 Collection and Processing of Network Pharmacology Data

The transcriptomic dataset GSE213621 was acquired from the gene expression omnibus (GEO) database (Kuang et al. 2023), comprising 68 healthy controls and 94 F3/F4‐stage NAFLD patients (Matsumoto et al. 2024). Data processing employed the sva package for integration, batch correction, and normalization. Differential expression analysis via limma identified significant genes (adj. *p* < 0.05, |log FC| > 0.585) (Zou et al. 2023), visualized through pheatmap and ggplot2 (Kuang et al. 2023).

### 2.10 Functional Enrichment and PPI Analysis

Functional enrichment of protein–protein interaction (PPI)‐derived NAFLD candidate (Li et al. 2022) genes was performed using clusterProfiler (Liu et al. 2020), with GO annotation covering biological processes, molecular functions, and cellular components, and KEGG analysis identifying associated signaling pathways (Kuang et al. 2023).

PPI network was constructed for NAFLD‐associated genes using STRING (confidence score > 0.4) and visualized in Cytoscape, with nodes representing proteins and edges indicating interactions (Kuang et al. 2023).

### 2.11 Machine Learning

Potential NAFLD diagnostic biomarkers were further screened by applying both LASSO logistic regression (glmnet) and random forest algorithms to candidate genes. Key genes were selected through both machine learning approaches, and the overlapping genes between the two methods were designated as the final set of candidate diagnostic markers (Hu et al. 2024).

Diagnostic performance of candidate genes was assessed across training and validation sets via receiver operating characteristic curve (ROC) analysis (pROC package), with area under the curve (AUC) > 0.7 indicating potential clinical relevance (Hu et al. 2024).

### 2.12 Molecular Docking

Molecular docking was performed using GAF as the ligand and the diagnostic candidate genes (identified through machine learning) as the corresponding receptors. Structural data for molecular docking were acquired from public databases: GAF (TCMSP), receptor information (UniProt), and 3D structures (PDB) (Hu et al. 2024).

Receptor proteins were prepared in PyMOL by eliminating water molecules, unnecessary ligands, and other contaminants. Ligand and processed receptors were converted to PDBQT format in AutoDock tools, followed by docking simulations with AutoDock Vina and result visualization in PyMOL (Hu et al. 2024). Binding affinities were evaluated based on docking energies, where values below −5 kcal/mol were considered indicative of significant ligand–receptor interactions.

### 2.13 Detection of EEF1A2, PPAR‐α, and p‐AMPK by ELISA

The protein expression levels of eukaryotic translation elongation factor 1 alpha 2 (EEF1A2) (Fine Test, Wuhan, China), peroxisome proliferator‐activated receptor alpha (PPAR‐α) (Fine Test, Wuhan, China), and phosphorylated AMP‐activated protein kinase (p‐AMPK) (RUIXIN BIOTECH, Quanzhou, China) were quantified using enzyme‐linked immunosorbent assay (ELISA) kits following the manufacturer's protocols. Briefly, after sample addition, incubation, washing, and color development, the absorbance was measured at 450 nm using a microplate reader. All measurements were performed in triplicate.

### 2.14 Statistical Methods

Data represent mean ± SD from three independent replicates (Kuang et al. 2023). Statistical significance (*p* < 0.05) was determined by one‐way ANOVA with least significant difference (LSD) test using SPSS 27.0 (Li et al. 2025). RSM was designed using Design‐Expert 13 software, and graphical representations were created with Origin 2021 (OriginLab, USA).

## 3 Results

### 3.1 Fermentation Conditions for the Highest GAF Yield

**FIGURE 1:**
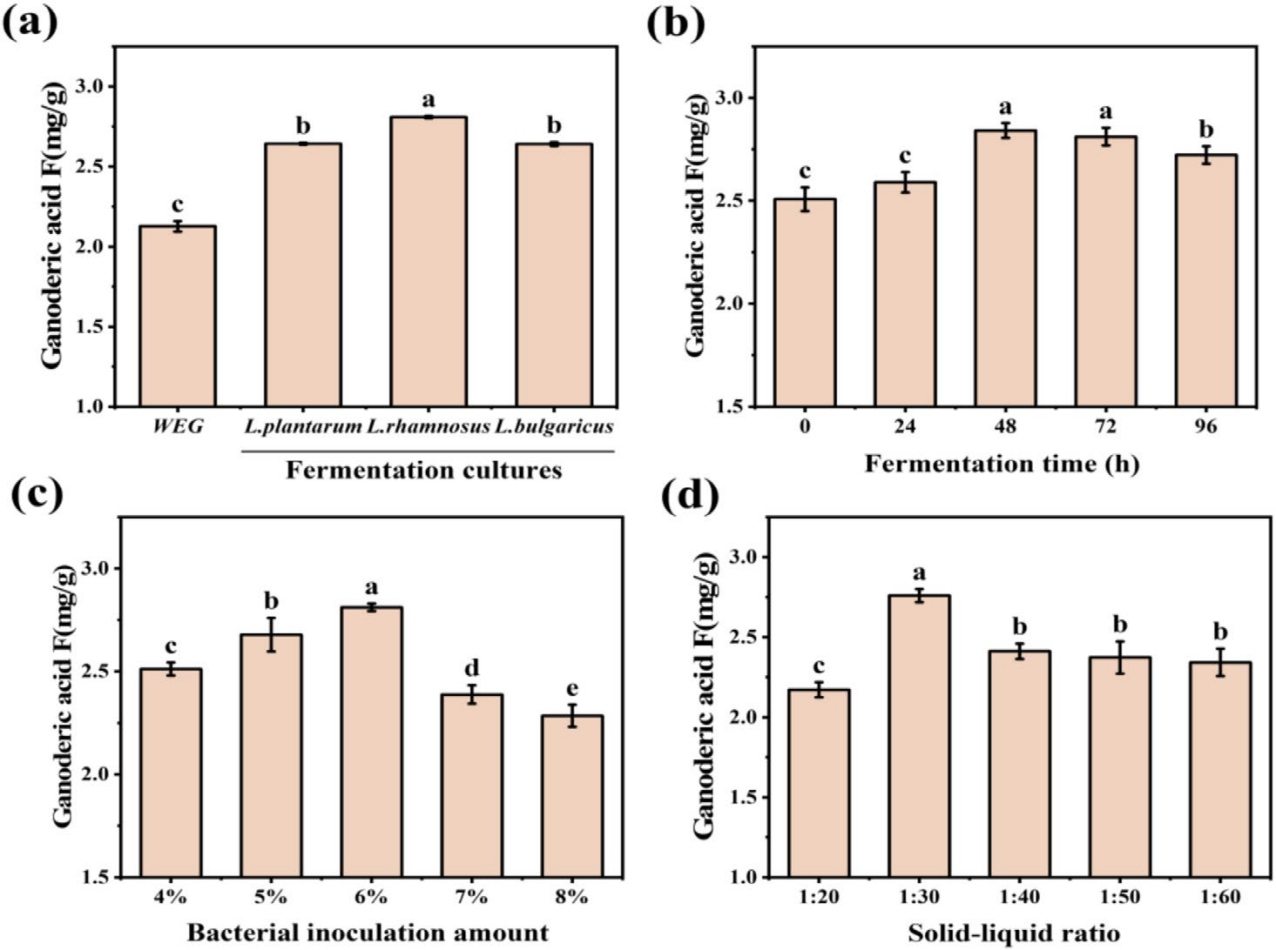
Effect of single‐factor experiments on the content of ganoderic acid F (*n* = 3). (a) Different strains, (b) fermentation times, (c) bacterial inoculation amount, (d) solid–liquid ratio. Significant differences (*p* < 0.05) are indicated by different letters in this and subsequent figures. Different letters denote significant differences between groups (*p* < 0.05), while the same letter indicates no significant difference.

**FIGURE 2:**
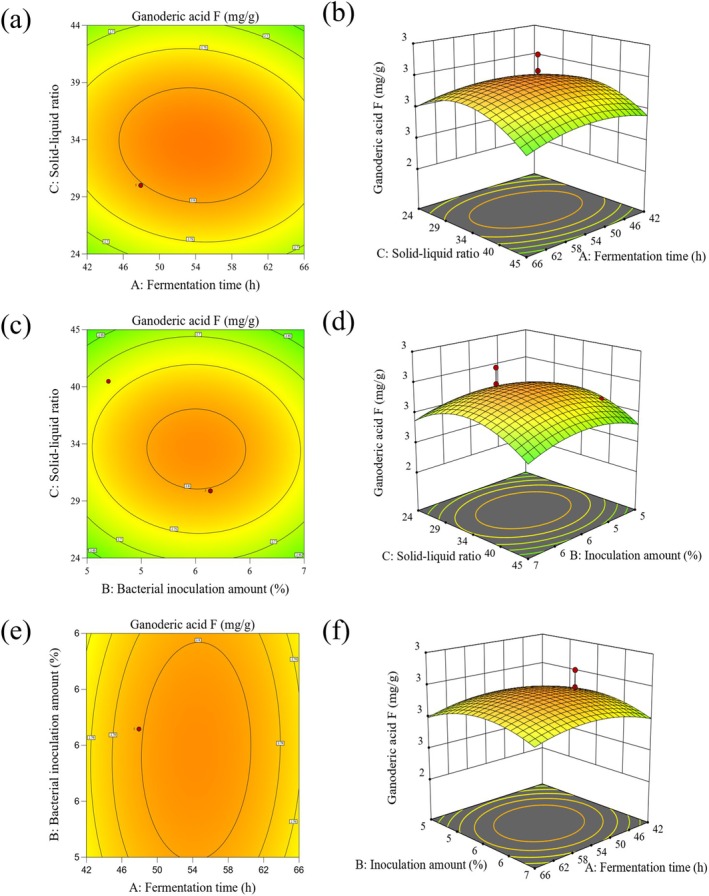
Response surface and contour plots visualize variable effects on ganoderic acid F production. (a, b) Fermentation time (A) and solid–liquid ratio (C). (c, d) Bacterial inoculation amount (B) and the solid–liquid ratio (C). (e, f) Fermentation time (A) and bacterial inoculation amount (B).

Among the three probiotic strains tested, *Lactobacillus rhamnosus* produced the highest GAF content (2.81 mg/g) in Figure 1a. Regarding fermentation time, GAF peaked at 48 h (2.84 mg/g), corresponding to the exponential phase of bacterial growth. Beyond this point, nutrient depletion and metabolic by‐products reduced enzyme activity, leading to a gradual decline (Figure 1b). Optimal bacterial inoculation was 6%, balancing growth rate and nutrient availability. Lower inoculation limited enzyme output, while higher inoculation caused early nutrient exhaustion and reduced conversion efficiency (Figure 1c). A 1:30 solid–liquid ratio maximized GAF yield by optimizing substrate concentration, oxygen transfer, and enzymatic activity (Figure 1d). Single‐factor experimental results identified fermentation time (A), inoculum size (B), and solid–liquid ratio (C) as critical parameters for a three‐level RSM design. A quadratic regression model was established using Design‐Expert 13 to evaluate their effects on GAF content (Tables S4 and S5). Analysis of interaction between factors (Figure 2), the interaction between fermentation time (A) and solid–liquid ratio (C) forms a pronounced curved surface with a peak in the center, indicating that optimal levels of both factors maximize GAF content.

### 3.2 Fermentation Increased the Levels of Active Compounds

**FIGURE 3:**
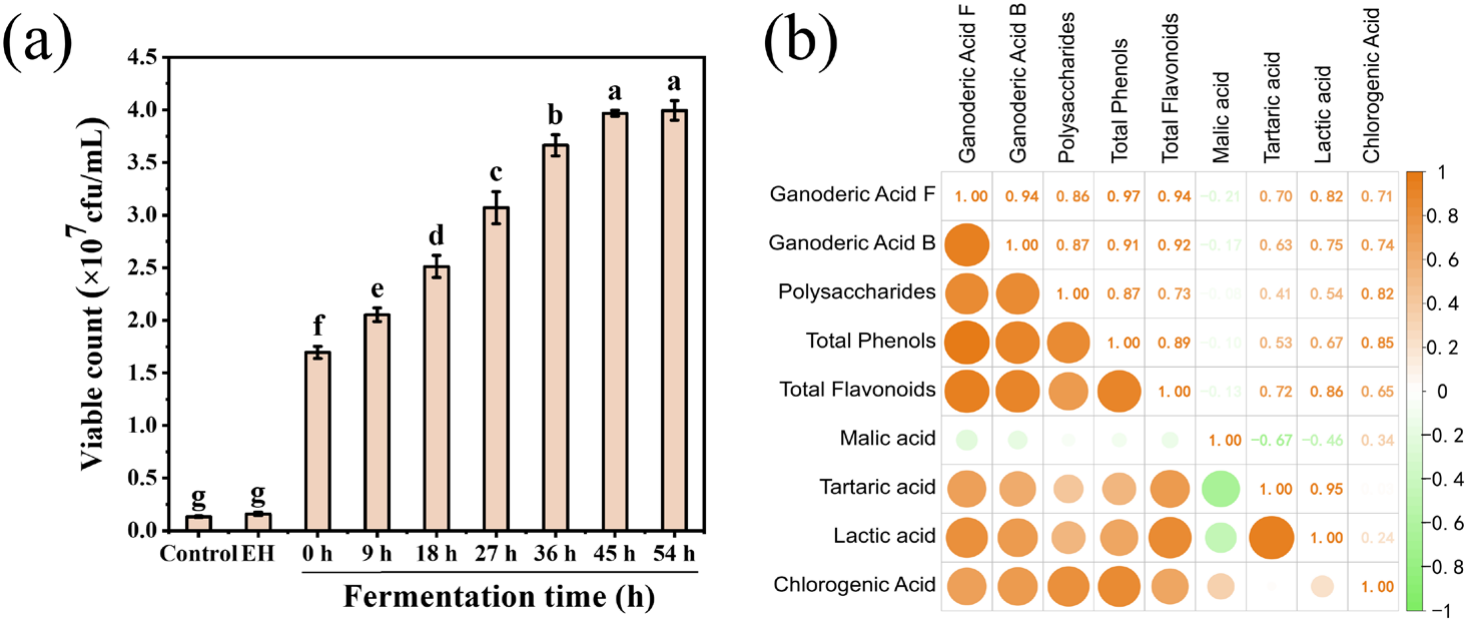
Analysis of active components at the optimal fermentation time (*n* = 3). (a) Viable count during fermentation. (b) Correlation heatmap of substance components in FBG and WEG liquids. Different letters denote significant differences between groups (*p* < 0.05), while the same letter indicates no significant difference.

Using water‐extracted *Ganoderma lucidum* (WEG) as the control and fermented *Ganoderma lucidum* broth (FBG) as the treatment, physicochemical indicators were compared to assess changes in key components such as ganoderic acids, organic acids, and phenols (Table 1). Compared with WEG, FBG showed significant increases in most measured substances. The viable count increased from 1.70 ± 0.06 × 10^7^ CFU/mL at 0 h to 3.97 ± 0.03 × 10^7^ CFU/mL at 45 h and remained stable through 54 h, indicating that the bacteria entered the stationary phase after 45 h (Figure 3a). The correlation heatmap analysis revealed strong positive correlations among ganoderic acids, total phenols, and total flavonoids, while malic acid exhibited negative correlations with tartaric acid and lactic acid (Figure 3b).

**TABLE 1:** Effect of synergistic fermentation with bacteria and enzymes on physicochemical indicators.

Component	WEG (mg/g)	FBG (mg/g)
Ganoderic acid F	2.11234 ± 0.0693	2.89237 ± 0.0298***
Ganoderic acid B	0.50073 ± 0.0111	0.75764 ± 0.0025***
Total phenols	3.22471 ± 0.0378	5.32742 ± 0.14468***
Total flavonoids	0.65661 ± 0.1106	1.8159 ± 0.0420***
Malic acid	22.16131 ± 1.0008	22.40184 ± 0.9134
Tartaric acid	21.24987 ± 0.7159	34.40234 ± 0.8157***
Lactic acid	12.40617 ± 0.7168	53.51164 ± 2.0037***
Chlorogenic acid	0.39286 ± 0.0071	0.61174 ± 0.0219***

*** *p* < 0.001 denotes a statistically highly significant difference.

### 3.3 FBG Ameliorated Lipid Accumulation in Liver Cells

**FIGURE 4:**
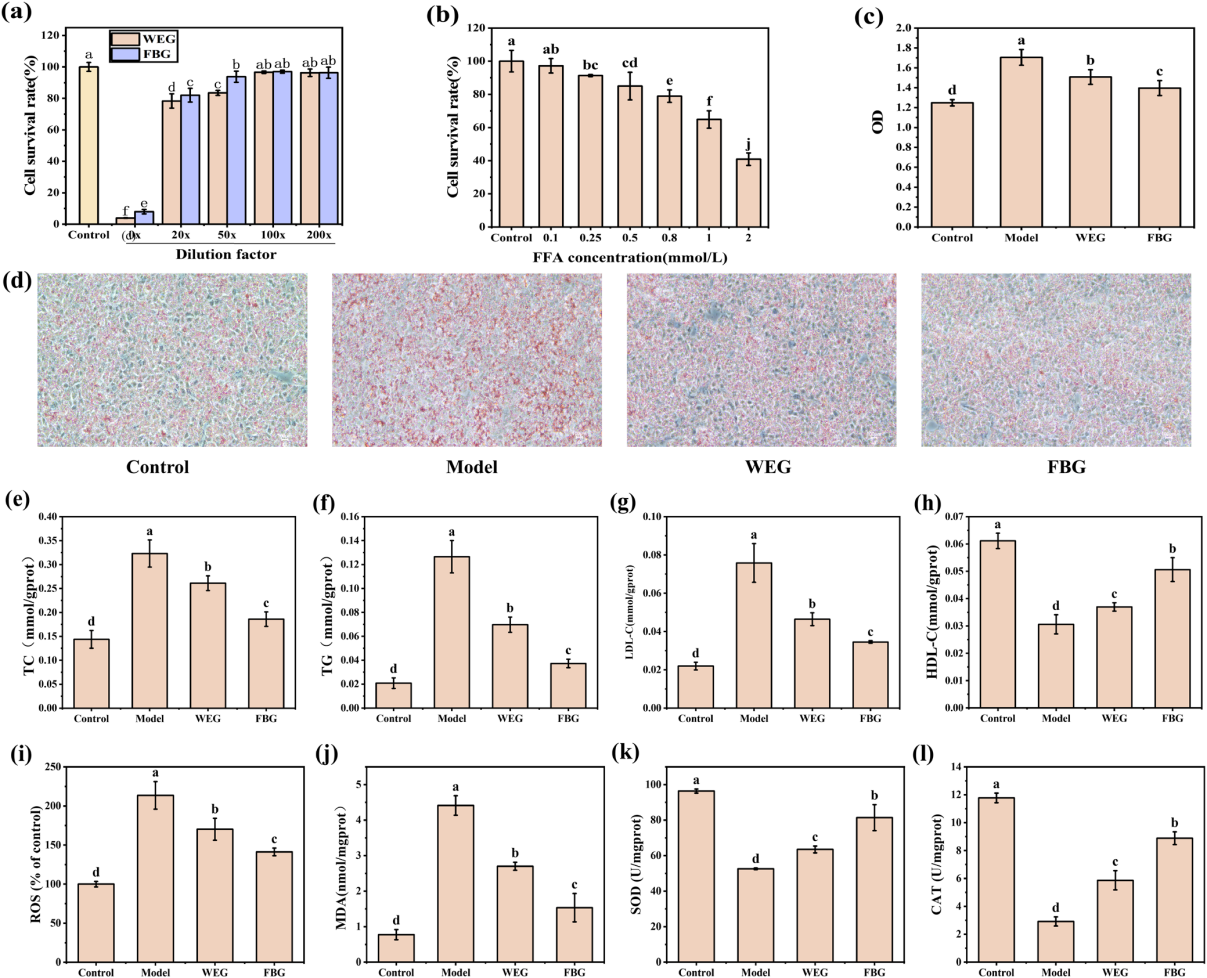
Effect of FBG on ameliorating NAFLD (*n* = 3). Control (no treatment), model (FFA‐induced), and treatment groups (WEG or FBG). (a) Cytotoxicity assay. (b) NAFLD cell model. (c) Relative content of lipids. (d) Visualization of intracellular lipid droplets by Oil Red O staining. (i–l) Intracellular contents of TC, TG, LDL‐C, and HDL‐C. (e–h) Antioxidant enzymes: ROS, MDA, SOD, CAT. Different letters denote significant differences between groups (*p* < 0.05), while the same letter indicates no significant difference.

FBG and WEG treatments (100×, 200×) showed no significant cytotoxicity in HepG2 cells versus control (no treatment) (*p* > 0.05) (Figure 4a). Cell viability assay showed that with the increase of FFA concentration, cell viability decreased in a dose‐dependent manner (Figure 4b). FFA treatment (0.1 mmol/L) was selected to establish the HepG2 lipid accumulation model. Relative to the control group (no treatment), the model group showed an increase in the accumulation of intracellular lipid droplets. Treatment with FBG markedly reduced lipid accumulation, exhibiting a stronger effect than WEG (Figure 4c,d).

TC (Figure 4e) and TG (Figure 4f) levels were significantly elevated in the model group compared with other groups (*p* < 0.05). Both FBG and WEG effectively reduced intracellular TC and TG, while FBG achieved significantly greater reductions than WEG. LDL‐C, which transports cholesterol from the liver to peripheral tissues, was markedly higher in the model group (Figure 4g). Both FBG and WEG significantly decreased LDL‐C levels, with FBG showing a superior effect compared to WEG (*p* < 0.05). HDL‐C levels were significantly reduced in the model group (*p* < 0.05) but restored by FBG and WEG treatments, with FBG showing superior efficacy (*p* < 0.05) (Figure 4h).

### 3.4 FBG Ameliorated NAFLD‐Induced Damage in HepG2 Cells

To assess the antioxidant capacity of FBG and WEG, intracellular ROS levels were measured (Figure 4i). The control group exhibited a baseline ROS level of 100% ± 3.45%. Upon FFA induction, the ROS level in the model group increased significantly. Treatment with 100× diluted FBG and WEG significantly lowered intracellular ROS versus the model group (*p* < 0.05).

The control group showed a low malonic dialdehyde (MDA) level (0.78 ± 0.14 nmol/mg protein), which significantly increased in the model group (4.41 ± 0.27 nmol/mg protein) (Figure 4j). Both FBG and WEG treatments significantly reduced MDA content (*p* < 0.05), with FBG showing a stronger effect. After FFA induction (Figure 4k,l), SOD activity decreased to 52.57 ± 0.49 U/mg protein, and CAT activity dropped to 2.92 ± 0.32 U/mg protein. Treatment with FBG and WEG significantly increased both enzyme activities.

### 3.5 Screening and Functional Enrichment of NAFLD‐Related DEGs

**FIGURE 5:**
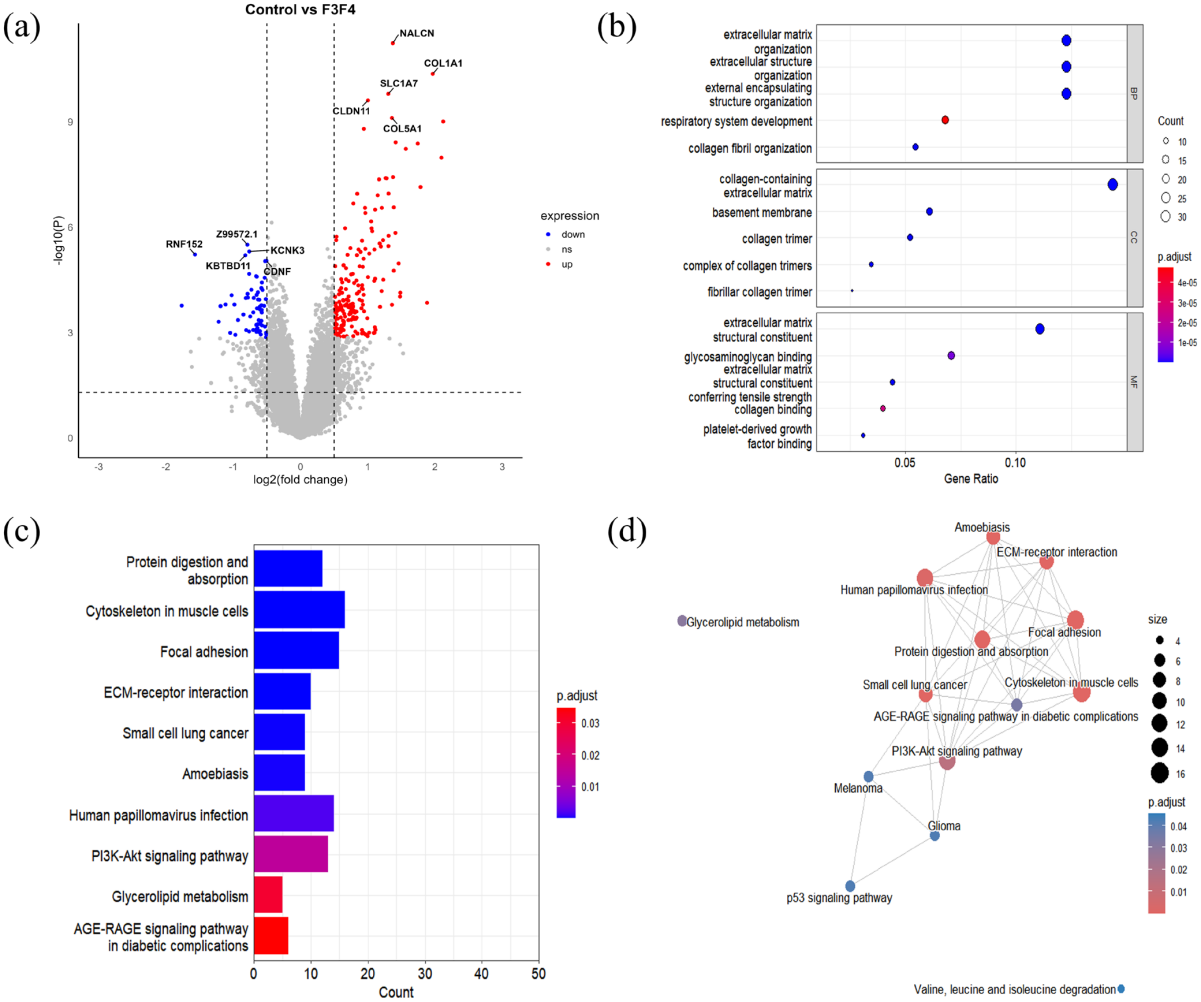
Bioinformatic analysis of NAFLD‐related genes. (a) Volcano plot of DEGs. (b) GO and (c) KEGG enrichment bubble plots (node size: Gene count); color intensity: ‐log10 (*p*.adjust). (d) PPI network of candidate driver genes.

Differentially expressed genes (DEGs) in GSE213621 were identified using limma (adj. *p* < 0.05). A total of 256 DEGs were detected in patient samples at the F3/F4 stage, including 193 upregulated and 63 downregulated genes (Figure 5a). Functional enrichment of NAFLD‐related DEGs was performed using the database for annotation, visualization and integrated discovery (DAVID). GO and KEGG analyses were performed to categorize the gene functions (Figure 5b–d). The results revealed significant enrichment in extracellular matrix organization and structure, collagen‐associated cellular components, and molecular binding, highlighting the critical role of extracellular matrix‐related processes and molecular interactions in NAFLD.

### 3.6 Identification of Diagnostic Biomarkers via Machine Learning

**FIGURE 6:**
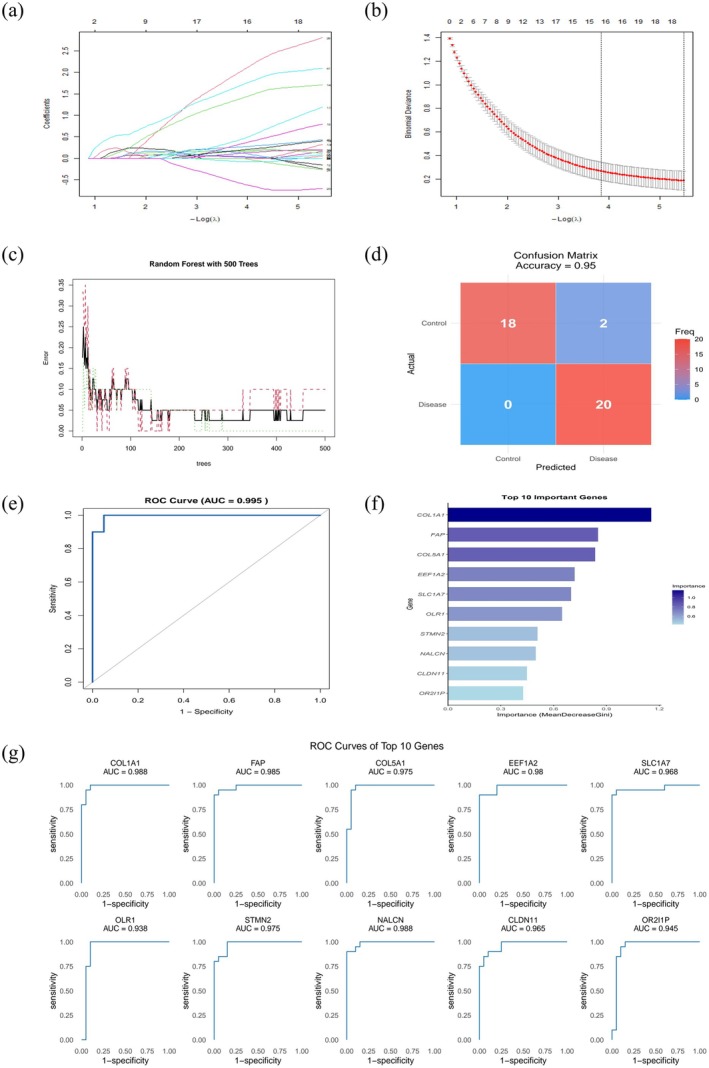
Machine learning‐based identification of diagnostic biomarkers. (a, b) Determination of the optimal lambda parameter and minimum error value for selecting diagnostic biomarkers via the LASSO logistic regression algorithm. (c) Error variation of the random forest model with 500 trees. (d) Confusion matrix plot of the NAFLD control group and disease group. (e) ROC curve. (f) Bar chart of the top 10 important genes. (g) ROC curves of the top 10 important genes.

The random forest (RF) algorithm ranked gene importance using the mean decrease gini index, screening the top 10 disease‐associated important genes as potential pathogenic factors. Concurrently, LASSO regression identified 20 candidate genes (Figure 6a) for the diagnostic model.

The coefficient path plot shows changes in gene coefficients with varying regularization strength (−Log(*λ*)), reflecting gene weight regulation and supporting core gene selection via LASSO. The binomial deviance plot (Figure 6b) indicates decreasing deviance stabilizing as regularization weakens, aiding optimal parameter selection and model balance. In the RF error trend plot (Figure 6c), model error decreased rapidly with increasing trees and stabilized around 200 trees, demonstrating model convergence and stability.

The diagnostic model based on the 20 core genes achieved an accuracy of 0.95 (Figure 6d). Among control samples, 18 were correctly classified, and 2 were misclassified as diseased. ROC curve analysis was performed on each gene and on the combined predictive model to assess their diagnostic performance. The results showed the ROC curve of the combined model lies above the diagonal reference line (AUC = 0.5) and approaches the top‐left corner (AUC = 0.995), reflecting optimal sensitivity and specificity across various thresholds (Figure 6e). Additionally, the top 10 important genes identified by random forest analysis were ranked based on their mean decrease gini values (Figure 6f,g), with Collagen type I alpha 1 chain (COL1A1) having the highest importance, followed by fibroblast activation protein (FAP), collagen type V alpha 1 chain (COL5A1), and others in descending order.

### 3.7 Molecular Docking Validation

**FIGURE 7:**
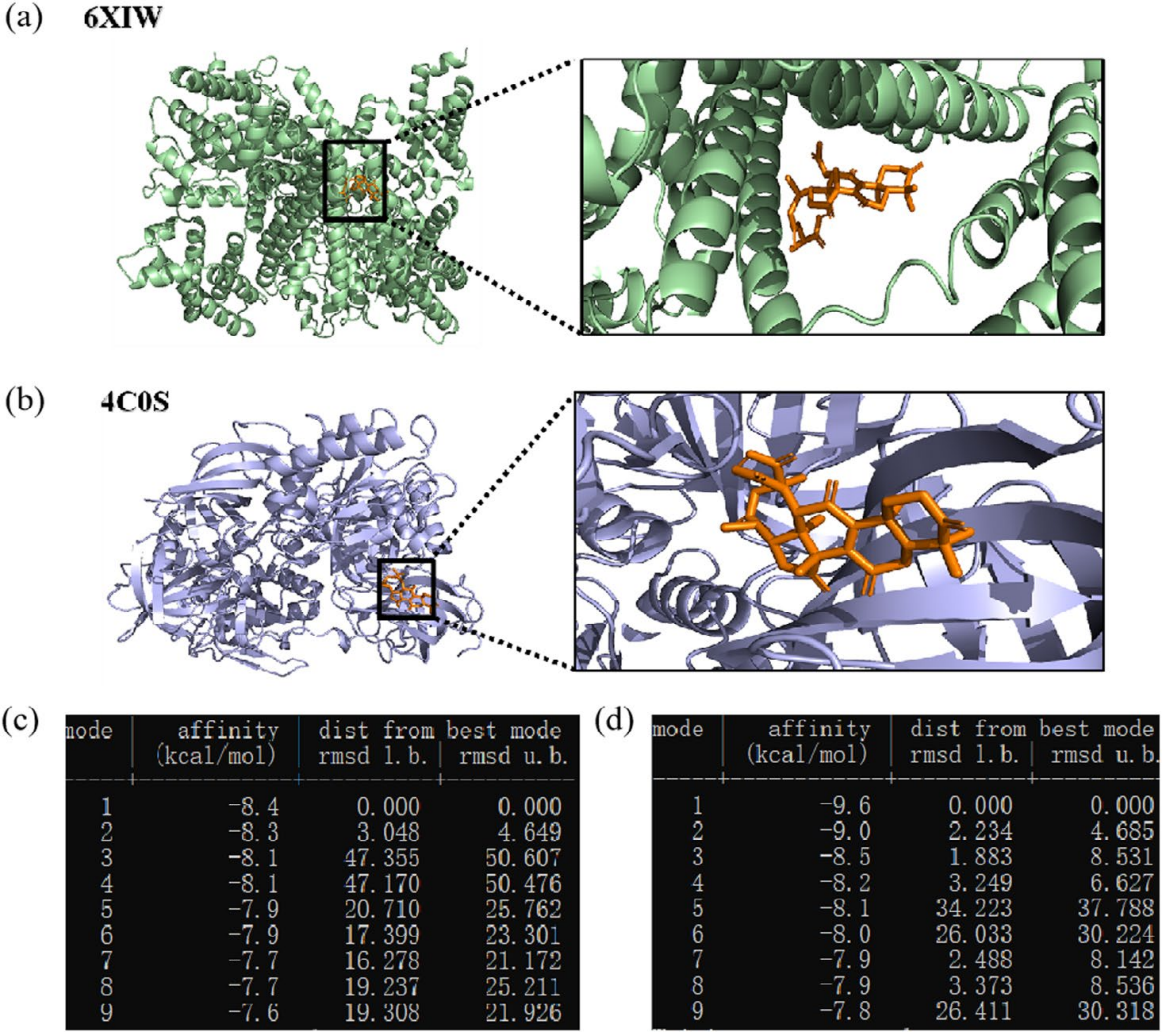
Molecular docking of NALCN and EEF1A2 with ganoderic acid F. (a) Molecular docking of NALCN (PDB ID: 6XIW) with ganoderic acid F. (b) Molecular docking of EEF1A2 (PDB ID: 4COS) with ganoderic acid F. (c) Molecular binding energy of 6XIW. (d) Molecular binding energy of 4COS.

The common overlapping genes (sodium leak channel, nonselective (NALCN) with PDB ID: 6XIW and EEF1A2 with PDB ID: 4COS) were screened from the top 10 important genes. They performed molecular docking analysis with GAF. Detailed information on the molecular binding is provided in Figure 7a–d. For the NALCN, the best mode (with a distance of 0 from itself) is mode 1, with an affinity of −8.4 kcal/mol (Figure 7c). For the EEF1A2, mode 1 has an affinity of −9.6 kcal/mol (Figure 7d), while other modes have slightly lower affinities and different root mean square deviation (RMSD) values relative to the best mode, reflecting differences in binding conformations. The results showed a high possibility for GAF to function through binding with NALCN or EEF1A2.

### 3.8 The Anti‐ Lipid Mechanism of GAF

**FIGURE 8:**
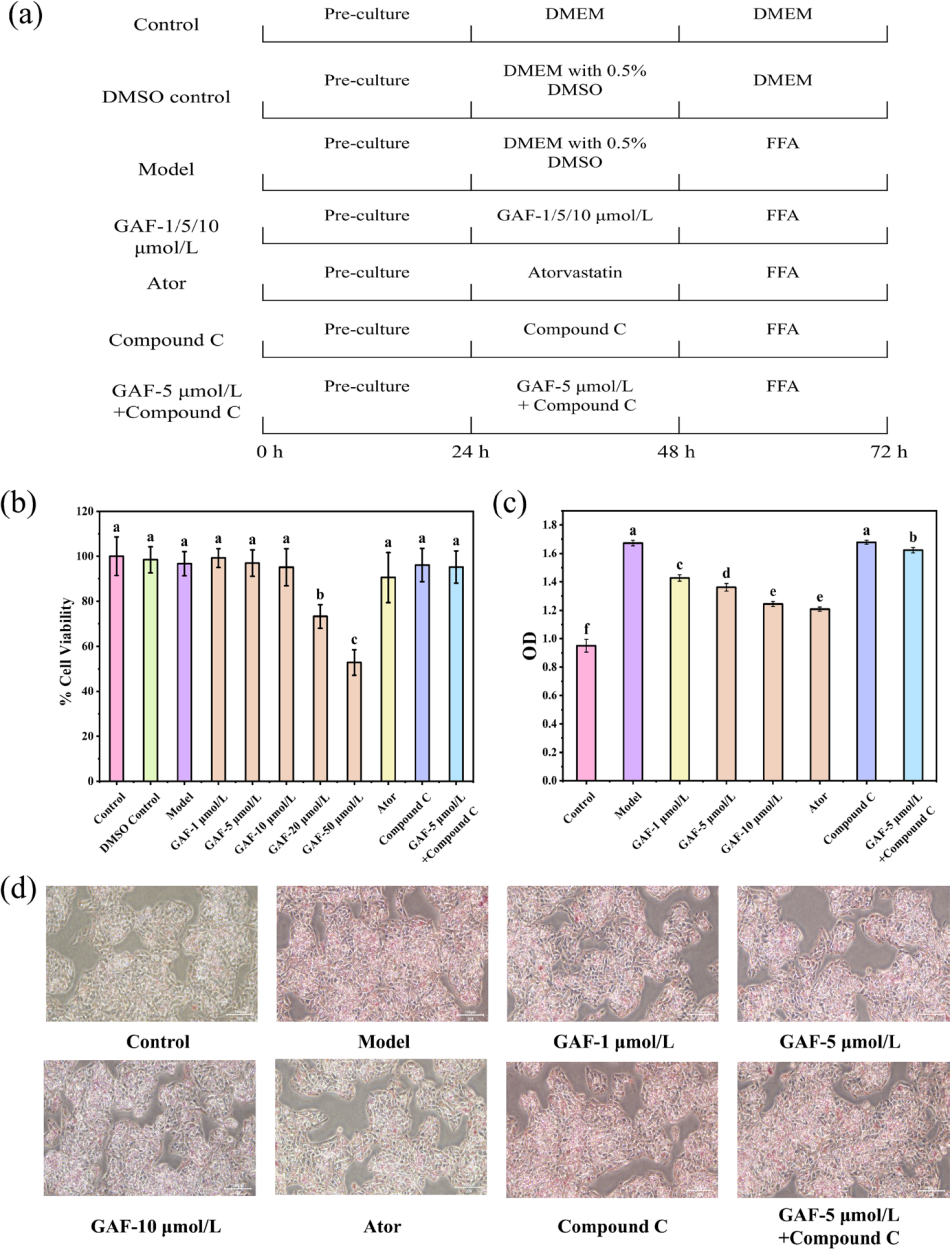
Effects of ganoderic acid F on cell viability and lipid accumulation in FFA‐induced HepG2 cells (*n* = 3). (a) Schematic diagram of experimental grouping. (b) Cell viability was assessed by MTT assay. (c) Lipid content was quantified by Oil Red O staining at OD_490_. (d) Representative images of Oil Red O staining (200× magnification). Different letters denote significant differences between groups (*p* < 0.05), while the same letter indicates no significant difference.

To further verify the results obtained from network pharmacology and molecular docking, an additional experiment was performed with GAF. The specific group design was exhibited in Figure 8a. Atovastatin was selected as positive control, while Compound C was used to explore the mechanism of GAF in AMPK pathway. The cell viability in the GAF‐20 and GAF‐50 μmol/L groups was significantly reduced (*p* < 0.05). Thus, GAF with the concentrations of 1, 5, and 10 μmol/L was selected as low, medium, and high doses for subsequent experiments (Figure 8b).

**FIGURE 9:**
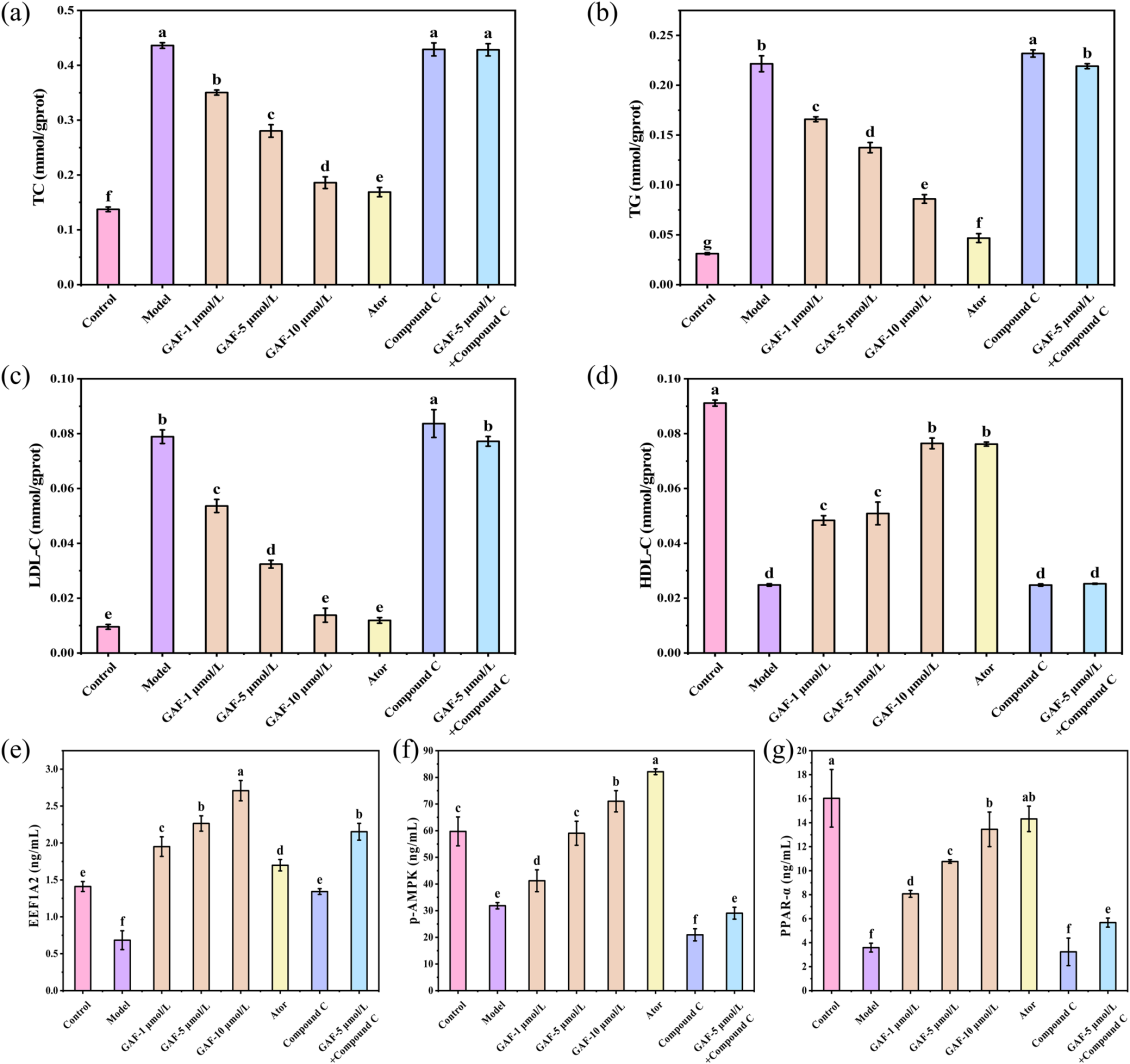
Effects of ganoderic acid F on lipid profile and protein expression in FFA‐induced HepG2 cells (*n* = 3). (a) Total cholesterol (T‐CHO) levels. (b) Triglyceride (TG) levels. (c) Low‐density lipoprotein cholesterol (LDL‐C) levels. (d) High‐density lipoprotein cholesterol (HDL‐C) levels. (e) EEF1A2 protein expression; (f) p‐AMPK protein expression. (g) PPAR‐α protein expression. Different letters denote significant differences between groups (*p* < 0.05), while the same letter indicates no significant difference.

The result of Oil Red O staining showed that the Model group had a significant increase in lipid content compared with the Control group. It indicated the successful establishment of the FFA‐induced lipid accumulation model. GAF treatment (1, 5, and 10 μmol/L) reduced lipid content in a dose‐dependent manner, while the GAF‐5 μmol/L + Compound C group exhibited significantly higher lipid content than the GAF‐5 μmol/L group, indicating that Compound C partially reversed the lipid‐lowering effect of GAF (Figure 8c,d). Comprehensive analysis of TC, TG, LDL‐C, and HDL‐C revealed that compared with the control group, the model group exhibited significantly elevated TC, TG, and LDL‐C levels and a significantly reduced HDL‐C level (*p* < 0.05). The GAF treatment (1, 5, and 10 μmol/L) reduced TC, TG, and LDL‐C levels in a dose‐dependent manner, while the atorvastatin positive control group showed comparable effects. Notably, the GAF‐5 μmol/L + Compound C group totally abolished its anti‐lipid effect compared with the GAF‐5 μmol/L group, indicating that the regulatory effect on lipid metabolism of GAF was possibly through the AMPK pathway (Figure 9a–d).

To further elucidate the pathway through which GAF exerted its lipid‐lowering effect, the EEF1A2, p‐AMPK, and PPAR‐α protein levels were tested with ELISA kits. The model group exhibited a significant decrease in EEF1A2 level, while GAF treatment improved EEF1A2 expression in a dose dependant manner, which proved the upregulatory effect of GAF for EEF1A2 level (Figure 9e). The GAF‐5 μmol/L + Compound C group had a similar level of EEF1A2 with the GAF‐5 μmol/L group, indicating that EEF1A2 was upstream of AMPK. In addition, GAF treatment also upregulated the p‐AMPK expression in a dose dependant manner. While GAF‐5 μmol/L + Compound C group was totally reversed the effect (Figure 9f), suggesting that the AMPK pathway was crucial for GAF performing anti‐lipid property. Furthermore, the result showed that GAF treatment could promote the expression of PPAR‐α, thus facilitating the lipid decomposition (Figure 9g). These findings further demonstrate that GAF ameliorates FFA‐induced lipid accumulation by activating the EEF1A2/p‐AMPK/PPAR‐α pathway.

## 4 Discussion

In this study, RSM was employed to optimize the bacteria‐enzyme synergistic fermentation process of *Ganoderma lucidum*. Previous studies have demonstrated that RSM can be effectively used to enhance the production of bioactive metabolites in *Ganoderma lucidum* (Shataer et al. 2026). Consistent with these reports, our results indicated that fermentation time had the most significant effect on GAF yield (*p* < 0.001), followed by solid–liquid ratio and inoculum size, with a significant interaction observed between fermentation time and solid–liquid ratio. This finding aligns with the study (Hu et al. 2017), which demonstrated through time‐course titration experiments that fermentation time critically influences ganoderic acid accumulation in *Ganoderma lucidum* submerged fermentation. Furthermore, the study (Tajik et al. 2024) employed RSM to optimize the submerged fermentation conditions of *Ganoderma lucidum*, determining the optimal medium formulation for ganoderic acid production and achieving a significant increase in ganoderic acid yield. Our results demonstrated that under the optimal conditions (54 h, 6% inoculum, 1:34 solid–liquid ratio), the actual yield of GAF (2.89 ± 0.03 mg/g) slightly exceeded the predicted value, indicating that the optimized process possesses good stability and predictability. Collectively, our results demonstrate that the RSM‐based optimization strategy provides a reliable approach for enhancing the production of GAF.

Regarding the effect of bacteria‐enzyme synergistic fermentation on the bioactive components of *Ganoderma lucidum*, previous studies demonstrated that laccase from *Ganoderma lucidum* acts synergistically with cellulase preparations, increasing total glucose yields by 17%–33% during enzymatic hydrolysis of lignocellulosic biomass (Sitarz 2013). Furthermore, *Lactobacillus rhamnosus GG* has been shown to secrete various extracellular enzymes, including peptidoglycan hydrolases (such as the Msp1/p75 protein), which possess peptidoglycan hydrolase activity and are capable of degrading bacterial cell wall structures (Lebeer et al. 2012). Our results demonstrated that the content of ganoderic acids F and B increased by approximately 37% and 51%, respectively, likely due to probiotic enzyme activity breaking down *Ganoderma* cell walls and converting bound precursors. Total phenols and chlorogenic acid increased by 65% and 56% (Boateng et al. 2024), while total flavonoids rose by 177%, which may be associated with cell wall disruption releasing bound phenolic and flavonoid compounds (Chen et al. 2011). Lactic acid increased by 331% (12.41 to 53.51 mg/g) and tartaric acid also increased by 62%, which may be produced by probiotics metabolizing sugars (Morales‐Landa et al. 2025). Malic acid levels remained nearly unchanged, which may suggest that it is not a major probiotic metabolite. Overall, bacteria‐enzyme synergistic fermentation may promote the release and transformation of ganoderic acids, phenols, and flavonoids by facilitating cell wall degradation.

The study demonstrated that daily supplementation of submerged *Ganoderma lucidum* culture significantly reduced plasma triglyceride levels and elevated HDL‐C levels in type 2 diabetic rats (Huang et al. 2020). Consistent with previous reports, our results demonstrated that FBG treatment significantly reduced intracellular TC, TG, and LDL‐C levels while elevating HDL‐C levels in FFA‐induced HepG2 cells, showing superior efficacy compared to WEG, suggesting that fermentation enhances the lipid‐lowering potential of *Ganoderma lucidum*. Fermented *Ganoderma lucidum* polysaccharides significantly decreased intracellular ROS and MDA contents, increased T‐AOC levels, and activated CAT, SOD, and glutathione peroxidase (GSH‐Px) activities in H_2_O_2_‐induced HepG2 cells (Zhao et al. 2023). Our results above indicate that FBG effectively mitigates lipid peroxidation, reducing MDA accumulation and alleviating cellular oxidative damage. Hepatic injury was assessed by measuring key antioxidant enzymes SOD and CAT. Therefore, FBG may potentially improve lipid accumulation. However, the current evidence is limited to in vitro studies, and further in vivo and clinical validation is needed.

To further explore the mechanism of GAF in NAFLD, DEGs in GSE213621 were analyzed. GO and KEGG analyses indicated that GO and KEGG enrichment analyses revealed that glycerolipid metabolism and the AGE‐RAGE signaling pathway exhibited the highest significance among all enriched pathways. Glycerolipid metabolism is a central pathway in lipid homeostasis and energy regulation, with emerging evidence highlighting its role in cardiometabolic diseases (Poursharifi et al. 2025). The advanced glycation end products—receptor for advanced glycation end products (AGE‐RAGE) interaction contributes to fat accumulation in the liver, leading to inflammation, fibrosis, insulin resistance, and other complications of fatty liver disease (Asadipooya et al. 2019). Molecular docking analysis revealed that GAF binds to NALCN and EEF1A2 with significant binding activity (binding energy < −5 kcal/mol). It has been reported that EEF1A2 may indirectly suppress lipogenesis by activating the AMPK signaling pathway (Zhang et al. 2024). Network pharmacology analyses were performed using computational tools to generate predictions for identifying potential mechanisms and key bioactive components. These predictions require further experimental validation in future studies.

Subsequent in vitro experiments were performed to validate this prediction and to investigate the potential mechanism by which GAF ameliorates FFA‐induced hepatic lipid accumulation. A recent study has shown that lanostane‐type triterpenes from *Ganoderma lucidum* exhibit significant lipid‐lowering activity in OA‐induced HepG2 cells, using network pharmacology and molecular docking to elucidate the underlying mechanisms (Hu et al. 2026). Consistent with our findings, GAF (1, 5, and 10 μmol/L) significantly reduced lipid accumulation in a dose‐dependent manner, as evidenced by Oil Red O staining and TC, TG, and LDL‐C levels. The lipid‐lowering effect of GAF at 10 μmol/L was comparable to that of atorvastatin. Notably, co‐treatment with the AMPK inhibitor Compound C partially reversed this effect, indicating the involvement of the AMPK pathway. Further mechanistic investigation revealed that GAF upregulated EEF1A2 expression, which was suppressed in the FFA‐induced model. Compound C did not significantly alter EEF1A2 expression, suggesting that EEF1A2 acts upstream of AMPK. Additionally, GAF enhanced AMPK phosphorylation and PPAR‐α expression, effects that were abolished by Compound C, confirming that AMPK is a central mediator of the lipid‐lowering effect of GAF. Recent studies support our finding that EEF1A2 acts upstream of AMPK. It was shown that eEF1A2 overexpression alleviated AMPK phosphorylation inhibition, an effect reversed by Compound C without altering EEF1A2 expression. Additionally, direct binding between AMPKα1 and EEF1A1 has been confirmed (Xing et al. 2026). Together, these findings support that GAF ameliorates FFA‐induced hepatic lipid accumulation via the EEF1A2/p‐AMPK/PPAR‐α pathway.

A key contribution of this study is the enhanced understanding of bacteria‐enzyme synergistic fermentation of *Ganoderma lucidum* fruiting bodies and its potential alleviating effects on NAFLD. An optimized fermentation process was established using RSM, which significantly increased the yields of GAF and other bioactive components. In FFA‐induced HepG2 cells, the fermented product FBG exhibited superior lipid‐lowering effects compared to the unfermented water extract. Through network pharmacology, molecular docking, and cellular mechanistic studies, GAF was identified as a key active component that alleviates hepatic lipid accumulation by activating the EEF1A2/p‐AMPK/PPAR‐α signaling pathway. These findings provide a molecular basis for the potential application of GAF in NAFLD intervention. It should be emphasized that the above experimental findings regarding the molecular pathways are based entirely on in vitro cell‐based assays and have not yet been validated by gene knockout or in vivo animal studies. Therefore, these mechanisms should be further investigated in future research.

Nevertheless, this study has several limitations. The lipid‐lowering effects of FBG and GAF were assessed only in HepG2 cells in vitro, which may not fully recapitulate the complex pathophysiology of NAFLD in vivo. Thus, further validation using animal models and clinical trials is required to confirm the therapeutic potential of FBG for NAFLD treatment.

## 5 Conclusion

This study demonstrates that the bacteria‐enzyme synergistic fermentation broth of *Ganoderma lucidum* (FBG) exhibits superior bioactivity compared to traditional water‐extracted preparations. FBG showed significantly enhanced contents of GAF, phenols, and flavonoids, along with improved antioxidant capacity. In NAFLD cell models, FBG effectively reduced lipid accumulation, oxidative stress markers (ROS and MDA), and enhanced antioxidant enzyme (SOD) activity. Network pharmacology and molecular docking showed that GAF binds favorably to NALCN and EEF1A2. In FFA‐induced HepG2 cells, GAF reduced lipid accumulation and oxidative stress, upregulated EEF1A2, enhanced AMPK phosphorylation, and increased PPAR‐α expression. The AMPK inhibitor Compound C partially reversed the lipid‐lowering effect and blocked p‐AMPK and PPAR‐α regulation without affecting EEF1A2, indicating that EEF1A2 acts upstream of AMPK. These results confirm the EEF1A2/p‐AMPK/PPAR‐α pathway as a key mediator in the potential amelioration of NAFLD‐related lipid disorders by GAF. However, these preliminary findings are based on cellular experiments, and further validation of efficacy and safety in animal models and clinical trials is required.

## Author Contributions

**Zhenzhen Wang:** formal analysis, validation, visualization. **Ruyi Sha:** conceptualization, funding acquisition, project administration, supervision. **Kecheng Li:** data curation, formal analysis, investigation, visualization. **Jianwei Mao:** conceptualization, funding acquisition, project administration, supervision. **Manhui Sun:** writing – review and editing, conceptualization, writing – original draft, visualization, validation, formal analysis, data curation. **Jing Dai:** formal analysis, validation, visualization. **Min Cai:** formal analysis, investigation, methodology, validation, writing – review and editing.

## Funding

This research was supported by the Chongqing Research Program of Technological Innovation and Application Development (CSTB2025TIAD‐qykjggX0181).

## Ethics Statement

The authors have nothing to report.

## Conflicts of Interest

The authors declare no conflicts of interest.

## Supporting information


**Table S1:** Factors and levels of response surface experiment design.
**Table S2:**. The gradient elution mode.
**Table S3:**. Calibration curve of the standard determined by HPLC.
**Table S4:**. Optimization scheme and results of response surface experiment.
**Table S5:**. Results of analysis of variance (ANOVA) for response surface.

## Data Availability

The data of this research result can be provided by the corresponding author upon reasonable request.
