# Supplementary material for: The Potential Alleviating Property Against NAFLD by Ganoderma lucidum With Bacteria‐Enzyme Synergistic Fermentation
Source: Food Sci Nutr. 2026 Jun 2;14(6):e71981. doi: 10.1002/fsn3.71981 (PMC13240248; doi:10.1002/fsn3.71981)
Supplement: Supplementary file 1 — Table S1: Factors and levels of response surface experiment design. Table S2:. The gradient elution mode. Table S3:. Calibration curve of the standard determined by HPLC. Table S4:. Optimization scheme and results of response surface experiment. Table S5:. Results of analysis of variance (ANOVA) for response surface. [file FSN3-14-e71981-s001.docx]

Table S1

Factors and Levels of Response Surface Experiment Design

| level | factor | | |
| --- | --- | --- | --- |
|  | A (Fermentation Time)/h | B (Bacterial inoculation amount)/% | C (Solid-Liquid Ratio） |
| -1 | 24 | 5 | 1:20 |
| 0 | 48 | 6 | 1:30 |
| 1 | 72 | 7 | 1:40 |

Table S2

The gradient elution mode

| Time (min) | CH_3_OH (%) | Water (contaaining0.01%f  ormic acid, %) |
| --- | --- | --- |
| 0 | 40 | 60 |
| 25 | 90 | 10 |
| 25.01 | 40 | 60 |
| 30 | 40 | 60 |

Table S3

Calibration curve of the standard determined by HPLC

| Components | Calibration Curve | R^2^ |
| --- | --- | --- |
| Ganoderic acid F | y = 8E+06x – 15451 | R² = 0.999 |
| Ganoderic acid B | y = 8E+06x – 17682 | R² = 0.999 |
| Malic acid | y = 507463x - 178846 | 0.9999 |
| Tartaric acid | y = 987710x - 344230 | 0.9999 |
| Lactic acid | y = 252119x - 65654 | 0.9984 |
| Chlorogenic acid | y = 53256x + 1806.3 | 0.9994 |

Table S4

Optimization Scheme and Results of Response Surface Experiment

| Number | Fermentation Time (h) | Bacterial inoculation amount (%) | Solid-Liquid Ratio | Ganoderic Acid F (mg/g) |
| --- | --- | --- | --- | --- |
| 1 | 72 | 6 | 20 | 2.5006 |
| 2 | 72 | 5 | 30 | 2.6539 |
| 3 | 48 | 6 | 30 | 2.7663 |
| 4 | 24 | 5 | 30 | 2.3858 |
| 5 | 48 | 5 | 20 | 2.5697 |
| 6 | 24 | 6 | 20 | 2.2588 |
| 7 | 48 | 7 | 40 | 2.6772 |
| 8 | 48 | 5 | 40 | 2.7343 |
| 9 | 72 | 6 | 40 | 2.6229 |
| 10 | 48 | 6 | 30 | 2.7214 |
| 11 | 24 | 7 | 30 | 2.3478 |
| 12 | 48 | 7 | 20 | 2.5266 |
| 13 | 48 | 6 | 30 | 2.7802 |
| 14 | 48 | 6 | 30 | 2.8163 |
| 15 | 24 | 6 | 40 | 2.4837 |
| 16 | 72 | 7 | 30 | 2.6661 |
| 17 | 48 | 6 | 30 | 2.9039 |

Table S5

Results of Analysis of Variance (ANOVA) for Response Surface

| Source of Variance | Sum of Squares | Degrees of Freedom | Mean Square | F-Value | *p*-Value | Significance |
| --- | --- | --- | --- | --- | --- | --- |
| Model | 0.4738 | 9 | 0.0526 | 14.8400 | 0.0009 | *** |
| A-Fermentation Time | 0.1170 | 1 | 0.1170 | 32.9700 | 0.0007 | *** |
| B-Bacterial inoculation amount | 0.0020 | 1 | 0.0020 | 0.5588 | 0.4791 |  |
| C-Solid-Liquid Ratio | 0.0548 | 1 | 0.0548 | 15.4600 | 0.0057 | ** |
| AB | 0.0006 | 1 | 0.0006 | 0.1769 | 0.6867 |  |
| AC | 0.0026 | 1 | 0.0026 | 0.7423 | 0.4175 |  |
| BC | 0.0000 | 1 | 0.0000 | 0.0137 | 0.9101 |  |
| A² | 0.2081 | 1 | 0.2081 | 58.6600 | 0.0001 | *** |
| B² | 0.0161 | 1 | 0.0161 | 4.5400 | 0.0706 |  |
| C² | 0.0498 | 1 | 0.0498 | 14.0400 | 0.0072 | ** |
| Residual | 0.0248 | 7 | 0.0035 |  |  |  |
| Lack of Fit | 0.0061 | 3 | 0.0020 | 0.4336 | 0.7409 | Not Significant |
| Pure Error | 0.0187 | 4 | 0.0047 |  |  |  |
| Total Regression | 0.4987 | 16 |  |  |  |  |

Note: Significance levels are indicated as follows: ****p* < 0.001, ***p* < 0.01, and **p* < 0.05.
